# Comparative proteomic profiling of newly acquired, virulent and attenuated *Neoparamoeba perurans* proteins associated with amoebic gill disease

**DOI:** 10.1038/s41598-021-85988-8

**Published:** 2021-03-25

**Authors:** Kerrie Ní Dhufaigh, Eugene Dillon, Natasha Botwright, Victor Birlanga, Anita Talbot, Ian O’Connor, Eugene MacCarthy, Orla Slattery

**Affiliations:** 1 Marine and Freshwater Research Centre, Galway-Mayo Institute of Technology, Co. Galway, Ireland; 2 Conway Institute, University College Dublin, Co. Dublin, Ireland; 3 CSIRO Agriculture and Food, Livestock and Aquaculture, Queensland Biosciences Precinct, 306 Carmody Road, Brisbane, QLD 4067 Australia; 4 Microbiology Department, School of Natural Sciences, National University of Ireland Galway, Co. Galway, Ireland; 5 Department of Biopharmaceutical and Medical Science, Galway-Mayo Institute of Technology, Co. Galway, Ireland

**Keywords:** Proteome, Ichthyology

## Abstract

The causative agent of amoebic gill disease, *Neoparamoeba perurans* is reported to lose virulence during prolonged in vitro maintenance. In this study, the impact of prolonged culture on *N. perurans* virulence and its proteome was investigated. Two isolates, attenuated and virulent, had their virulence assessed in an experimental trial using Atlantic salmon smolts and their bacterial community composition was evaluated by 16S rRNA Illumina MiSeq sequencing. Soluble proteins were isolated from three isolates: a newly acquired, virulent and attenuated *N. perurans* culture. Proteins were analysed using two-dimensional electrophoresis coupled with liquid chromatography tandem mass spectrometry (LC–MS/MS). The challenge trial using naïve smolts confirmed a loss in virulence in the attenuated *N. perurans* culture. A greater diversity of bacterial communities was found in the microbiome of the virulent isolate in contrast to a reduction in microbial community richness in the attenuated microbiome. A collated proteome database of *N. perurans*, Amoebozoa and four bacterial genera resulted in 24 proteins differentially expressed between the three cultures. The present LC–MS/MS results indicate protein synthesis, oxidative stress and immunomodulation are upregulated in a newly acquired *N. perurans* culture and future studies may exploit these protein identifications for therapeutic purposes in infected farmed fish.

## Introduction

*Neoparamoeba perurans* is an ectoparasitic protozoan responsible for the hyperplastic gill infection of marine cultured finfish referred to as amoebic gill disease (AGD)^1^. Originally described in Tasmania in the 1980s^2^, AGD now has a global impact on susceptible finfish farms^3,4^ and is regarded as one of the most economically damaging, parasitic diseases of these farms^5^. The disease has a variety of hosts^6,7^; however susceptibility has been reported in the marine grow-out phase of *Salmo salar L*. farms^7,8^. Curative measures rely on the use of freshwater or hydrogen peroxide baths as persistent chronic infections may lead to fatalities; however, reoccurrence of the disease is common^9^. Clinical criteria for the diagnosis of AGD include infected gill tissue that displays numerous raised white mucoid lesions^10^ with over production of mucus from the host^11^, as well as lethargy and reduced growth of moribund fish ^5^. Gill histopathology of AGD infected fish display hyperplasia and lamellar fusion^12,13^.

The *Neoparamoeba* genus are a group of well documented pathogenic amoebae known to cause infection in marine fish and invertebrates^14–18^. The virulence factors of *N. perurans* have not been defined; however the presence of an extracellular protein^19^ and molecules involved in host attachment^20–23^ are suspected to be key virulence factors associated with the disease. Long term cultivation of *N. perurans* results in the loss of parasitic virulence in vivo^19^ , offering a platform for comparative research of virulent and attenuated cultures of the parasite. Development of attenuated cultures may occur as the parasite adapts to long term cultivation in vitro*,* where gene expression may be altered to aid proliferation in the new environment^24^.

Free living amoebae are home to numerous amoeba-resisting microorganisms, comprising bacteria, viruses and fungi^25^. Initial evaluations of the bacterial community structure of *N. perurans* were derived from AGD positive gill samples^26,27^ with a common family of bacteria successfully identified, namely *Flavobacteriaceae,* however the discriminating genus differed in the two studies. The presence of *Pyschroserpens* spp. was found by Bowman and Nowak^26^, in contrast to the finding of Embar-Gopinath, et al.^27^ where *Winogradskyella* spp.was identified. Interestingly, *Winogradskyella* spp. nearest phylogenetic neighbour is *Psychroserpens burtonensis* ^28^ however, Embar-Gopinath, et al.^27^ suggest not identifying *Psychroserpens* spp. in their study may be due to its fastidious behaviour in culture. *N. perurans* isolate based microbiome characterisation was recently achieved by McCarthy, et al.^29^ and Benedicenti, et al.^30^. Several genera coupled to *Pseudomonas* spp.*, Marinomonas* sp. and *Flavobacterium* sp. were identified^29^. Polygonal and clonal isolates of *N. perurans* shared several genera of both gram positive and gram negative bacteria with significant differences in relative abundances between the three isolates^30^. Despite the speculation surrounding the role of *N. perurans* microbiome in AGD, no study exists that directly compares the microbiome of a long-term cultured *N. perurans* isolate to a virulent isolate.

In the present study, the microbiome of an attenuated and virulent isolate of *N. perurans*, both maintained at 16 °C, was characterised by 16S rRNA Illumina MiSeq sequencing to identify similarities and divergences between isolates. Furthermore, this collated information was used to inform the 'bacterial protein database' that was incorporated with an amoebozoa database and *N. perurans* specific database, into the Andromeda search engine of MaxQuant^31^ for protein identification from 2D PAGE. In addition, two phenotypes of *N. perurans* were investigated for differential virulence against naïve smolts to validate the attenuation of *N. perurans* virulence over time. These phenotypes were compared by characterising their soluble proteome using 2D PAGE, alongside a newly acquired culture of *N. perurans*, providing a proteomic timeline of differential virulence.

## Results

### In vivo challenge

**Figure 1:**
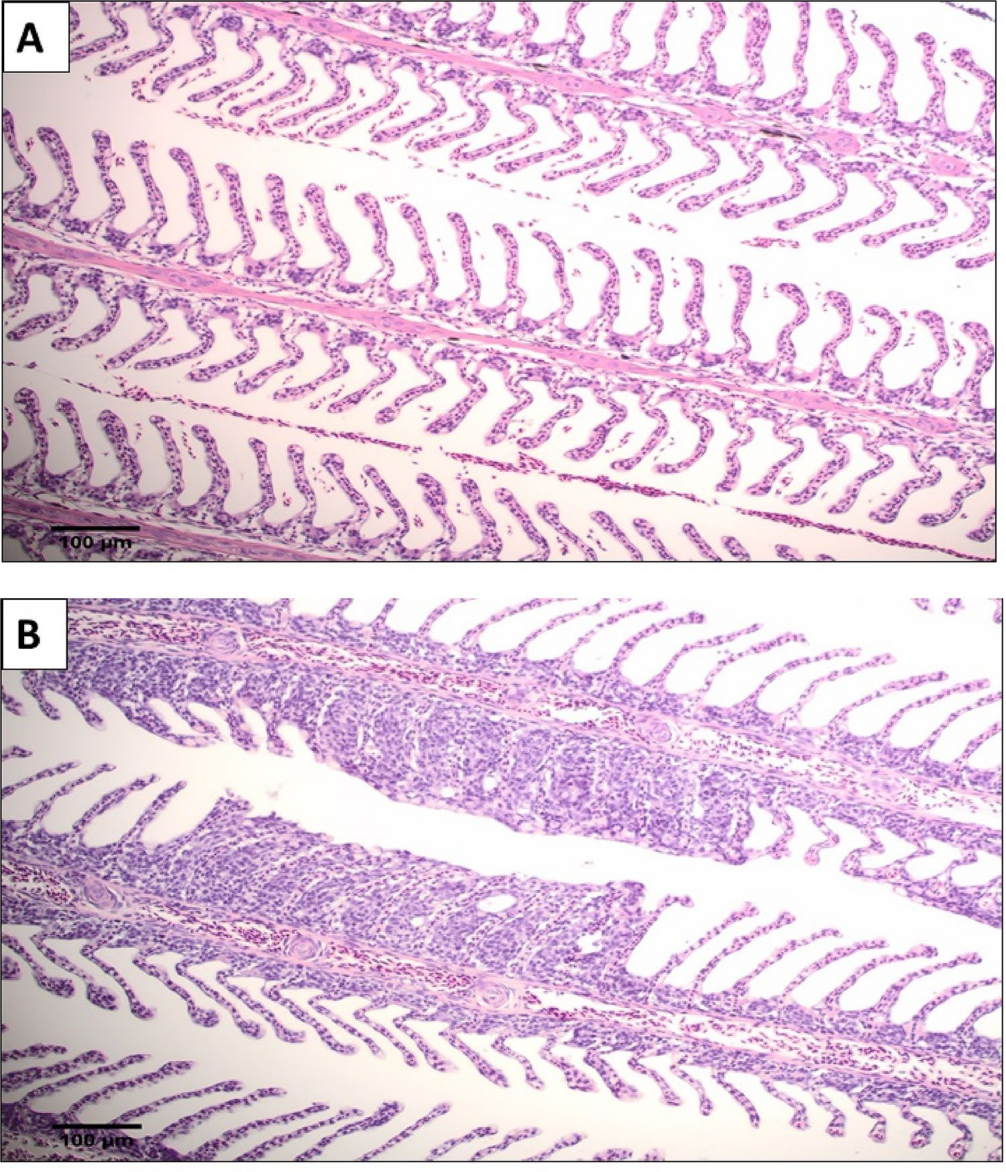
An example of H&E stained gill samples taken from non-AGD and AGD infected fish from the challenge trial. Scale bar = 100 µm. (**A**) Gill sample from the attenuated cohort. (**B**) Gill sample from the virulent cohort.

The control cohort remained negative for *N. perurans* for the duration of the trial, as confirmed by gill scoring, qPCR and histology. During gill scoring, both the control and attenuated cohort had no visible gross macroscopic lesions at 7 days post infection (dpi) and were assigned a gill score of 0, while two fish from the virulent cohort were gill score 1. One fish from the attenuated cohort and all fish from the virulent cohort sampled at 7 dpi were qPCR positive for *N. perurans*. Histological analysis revealed no pathology for fish sampled at 7 dpi. This observation is consistent with the early stage of the disease where gross gill pathology is not evident^32,33^, further strengthening the use of qPCR in identifying the presence of the parasite^32–34^. Gill scores > 1 were observed for each fish at 14 dpi for the virulent cohort, with qPCR analysis confirming the presence of *N. perurans* for each fish sampled. qPCR detected the presence of *N. perurans* in one fish sampled 14 dpi from the attenuated cohort, however no corresponding gills score or pathology was observed with this fish. Negative qPCR results were observed for each fish sampled in the attenuated cohort at 21 dpi. At 21 dpi, half the fish sampled from the virulent cohort were observed to have a gill score of 2 and the experiment was terminated. No AGD-like pathology was detected in the attenuated cohort (Fig. 1a). Histopathology for the virulent cohort revealed lamellar epithelium hyperplasia and fusion, with occasional formation of interlamellar lacunae (Fig. 1b).

### 16S Sequencing of attenuated and virulent *N. perurans* microbiome

**Figure 2:**
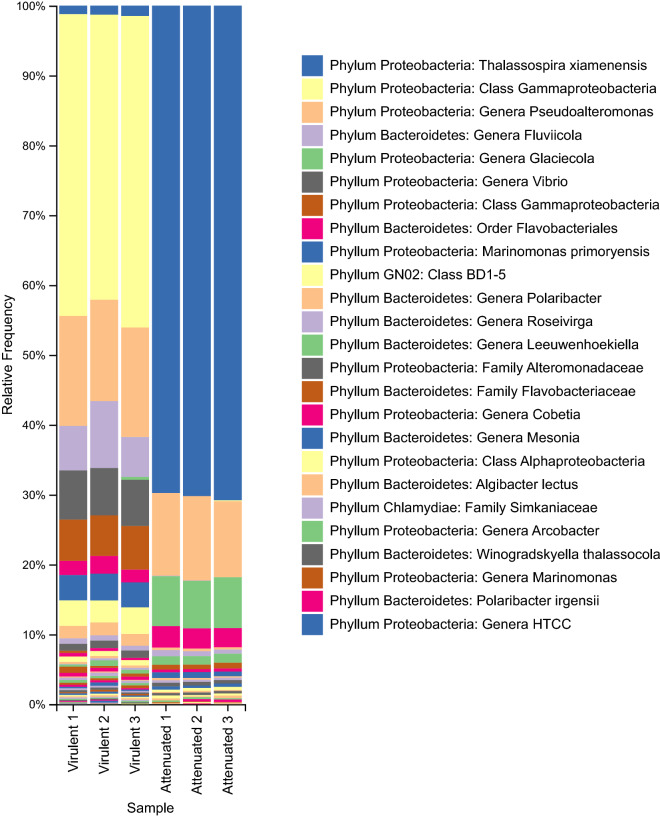
Relative abundance of the prokaryotic community in the attenuated and virulent cultures by 16S rRNA gene sequencing.

Relative abundances of OTUS (Fig. 2) were used to generate the resulting list of prominent taxa in both the attenuated and virulent *N. perurans* cultures. Gram negative bacteria were identified with Proteobacteria and Bacteroidetes being the dominant phyla in both cultures. A loss of microbial diversity and relative abundance is evident in the attenuated culture, presumably reflecting the lack of adaptation to prolonged culturing of these particular genera. This was most true for several identifications of Gammaproteobacteria in the virulent culture. Three independent identifications appeared only in the virulent culture suggesting the presence of this class warrants further investigation. An unknown Gammaproteobacteria had a higher relative abundance in the virulent culture and could potentially be implicated in AGD virulence. Differences in community structure were found in shifts of relative abundance of the species *Thalassospira xiamenensis* belonging to the class Proteobacteria from ~ 1% coverage in the virulent culture and ~ 70% coverage in the attenuated culture. The genus Vibrio was only identified in the virulent microbiome as was the phyla *Chlamydiae*, an interesting find due to both this genus and phylum’s role causing marine finfish disease. *Winogradskyella* was also identified in both microbiomes, however in much lower relative abundance than other genera. The UniProt proteomes of three genera from this sequencing analysis were selected for creation of the ‘bacterial’ protein database used in the proteomic characterisation of the *N. perurans* cultures in this study. These genera were: *Pseudoaltermonas, Fluviicola* and *Vibrio*. Additionally, due to the large coverage of *T. xiamenensis* in the attenuated, this species was also included in the database.

### 2DE and protein identification

**Figure 3:**
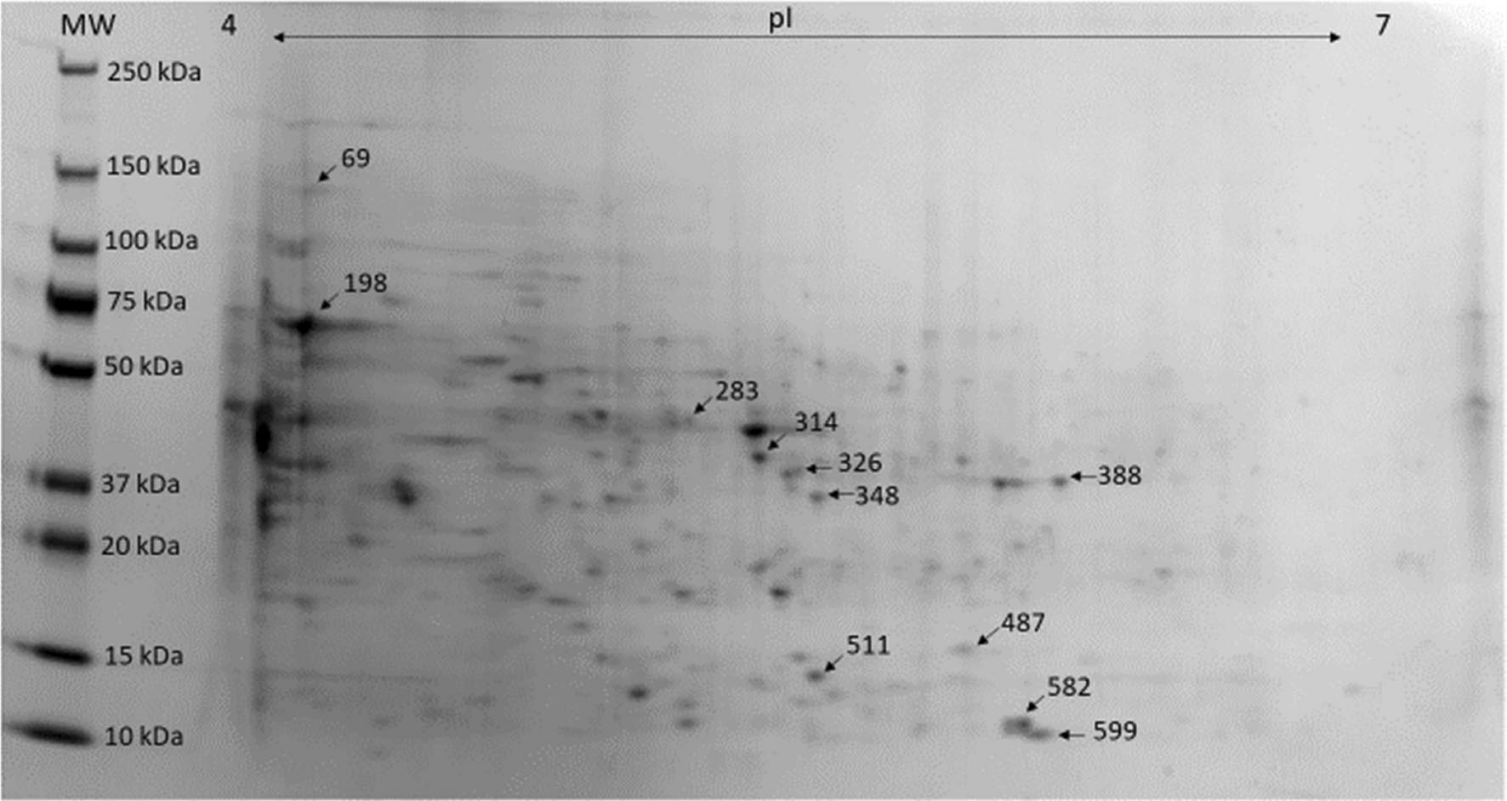
2D gel of the reference gel from the newly acquired culture of *N. perurans*. 11 spots displayed differential expression and were excised for LC MS/MS.

A total number of 98 spots identified by the Same Spots software were found to be statistically different in intensity, (*p* ≤ 0.05) across the three sets of replicate gels from the newly acquired, virulent and attenuated protein extracts. Eleven spots with a maximum fold change of ≥ 2 were chosen for further analysis using LC–MS (Fig. 3), resulting in the identification of 24 proteins (Supplementary Table S2), the majority derived from the *N. perurans* database (Table 1), 4 N*. perurans* proteins of which shared homology to proteins of other amoebozoa species (*Acanthamoeba spp*, *Planoprotostelium spp*) and 1 protein shared homology with *Thalassospira xiamenensis,* the gram negative bacterium largely evident in the attenuated microbiome analysis. Six proteins were not *N. perurans* associated, 3 proteins of which were bacterial and the remaining 3 proteins were from the Amoebozoa group. Thirteen proteins were exclusively assigned to *N. perurans* only, therefore the proteins identified in this study are predominantly amoebic in nature and furthermore, are from *N. perurans*. GeneBank IDs for *N. perurans* are provided in Supplementary Table S1. Ten of the 11 spots were found to be upregulated in the newly acquired culture, except for spot 69 which was more upregulated in the attenuated culture in comparison to both the virulent and newly acquired cultures. Protein expression was highest in the newly acquired culture, followed by the virulent and attenuated cultures from 10 of the 11 spots. Four of the protein spots were upregulated (*p* < 0.05) in the newly acquired culture followed by low expression in the attenuated culture and virulent cultures, however the change between the attenuated and virulent cultures was not statistically significant (*p* > 0.05). The identity of differentially expressed proteins is listed in Table 1, along with the corresponding intensity fold change and molecular weights. The function of these proteins can be broadly attributed to cellular proliferation, metabolism and immunomodulation. Some of the described proteins are involved in oxidative defence, plausibly protecting the parasite from the hostile host response.

**Table 1:** 2D spots with significant (*p* ≤ 0.05) fold changes from the newly acquired *N. perurans* culture identified by LC–MS/MS from *N. perurans*. The complete list of proteins identified from the large collated database containing *N. perurans* microbiome, Amoebozoa and *N. perurans* proteins are in Supplementary Table S2. Several proteins that are shared between *N. perurans* and species found in the bacteria and Amoebozoa database are distinguished in the organism header of Supplementary Table S2. *N. perurans* GenBank IDs can be found in Supplementary Table S1.

Estimated MW (kDa)	Estimated pI	Fold change	*p *value ≤ 0.05	Protein identification	No peptides matched	Coverage (%)	Biological function
20	5.61	4.6	0.005	ATP synthase subunit mitochondrial-like	2	6	Metabolism
20	5.61	4.6	0.005	Histone H2B	2	18.3	Protein synthesis
20	5.61	4.6	0.005	ADP-ribosylation factor 4 and 1	2	25.8	Cellular signalling
40	5.55	3	0.042	Heat shock protein 8	3	7.1	Stress response
70	4.45	2.5	9.55E−05	Peptidase C53 family protein	4	9	Immune evasion
70	4.45	2.5	9.55E−05	Carbohydrate ABC transporter substrate-binding protein	3	2.5	Metabolism
16	6.12	4.4	1.10E−04	Actin, cytoplasmic A3a isoform	8	22.6	Cytoskeleton
21	5.95	2.1	0.003	Cu–Zn Superoxide dismutase	2	16.3	Oxidative response
21	5.95	2.1	0.003	ADF-like domain-containing protein	2	15.5	Cytoskeleton
21	5.95	2.1	0.003	Lipoxygenase (lox) homology domain-containing protein 1	2	26.4	Cellular signalling
17	6.08	7.2	6.25E−04	Profilin conserved site domain-containing protein	6	64.3	Cytoskeleton, immunomodulation
17	6.08	7.2	6.25E−04	Profilin allergen	6	44.4	Cytoskeleton, immunomodulation
36	5.61	5.2	0.007	Malate dehydrogenase	4	13.8	Metabolism
45	5.36	1.9	0.033	Fragmin A	3	12.8	Cytoskeleton
45	5.36	1.9	0.033	Elongation factor 1	2	17.4	Cytoskeleton
45	5.36	1.9	0.033	Component of cytosolic 80S ribosome and 60S large subunit	2	7.7	Translation
45	5.36	1.9	0.033	Citrate synthase active	2	5.6	Metabolism
142	4.5	3.3	0.018	Aconitate hydratase	2	2.4	Oxidative stress

## Discussion

Attenuation of parasitic virulence has been attributed to long term cultivation in vitro that negatively impacts the organisms capacity to establish infection in the host model^35–37^. A complete loss of virulence has been previously reported during experimental in vivo trials using a clonal culture of *N. perurans* that was maintained in vitro for 3 years^19,38^. In the present study, a 3-year culture of *N. perurans* had a markedly reduced ability to establish AGD in a challenge trial using naïve salmon smolts, confirming attenuation of *N. perurans* virulence over time. Validation of virulence retention and loss in cultures was achieved using gill scoring, histopathology, and qPCR of gill samples from the in vivo AGD virulence challenge trial.

Gill scoring of six fish at each time point throughout the duration of the trial revealed no gill pathology for the attenuated cohort. In comparison, fish sampled in the virulent cohort were assigned cumulative gill scores at each time point until reaching the pre-determined humane endpoint. At 21 dpi three out of the six fish sampled were at a gill score of 2, resulting in the termination of the trial. No AGD-like pathology was detected in the control and attenuated cohort for the duration of the trial. Assessment of virulent cohort histology (Fig. 1) revealed the presence of AGD pathology with infected gills displaying lamellar epithelium hyperplasia and fusion^13,39^. qPCR assessment revealed that the majority of fish sampled in the virulent cohort were positive for *N. perurans*. In the attenuated cohort, only one in six fish sampled at each of 7 and 14 dpi was positive for *N. perurans* and all fish tested in this cohort were negative for the parasite at 21 dpi. These results confirm that using a combination of histology and qPCR is an invaluable approach in diagnosing AGD^32^, particularly in the early stage of the disease.

Due to the abundance of literature suggesting *N. perurans* microbiome may influence AGD progression^26,27,29,30^, 16S rRNA sequencing was employed to clarify the role of the microbiome in the virulent and attenuated isolates. Figure 2, clearly shows that time in prolonged culture results in microbial community shifts. A much broader and diverse group of bacteria were present within the virulent isolate. No gram-positive bacteria were identified in either isolate, in contrast to the study by Benedicenti, et al.^30^ where both gram positive and gram negative bacteria were identified in their polyclonal and two clonal cultures. Several gammaproteobacterial classes were found in the virulent isolate but were absent in the attenuated isolate. The presence of the genera *Vibrio* and the *Simkaniaceae* family warrants further investigation as these two microbes are causative agents of disease in marine shellfish and finfish aquaculture^40,41^.

Characterisation of the virulence molecules involved in AGD was achieved by comparing the proteomes of several *N. perurans* cultures. The soluble proteomic profile of a 3-year, 1-year and 70-day old *N. perurans* culture was analysed using 2D PAGE coupled with LC–MS/MS. The genome of *N. perurans* has recently been sequenced (Botwright, personal correspondence), enabling the comprehensive proteomic studies of *N. perurans* presented here. A total of 11 spots were excised from the newly acquired 2D PAGE gel (Fig. 3) based on the statistical criteria of ANOVA (*p* ≤ 0.05) and maximum fold change of ≥ 2 using the newly acquired culture as a reference. These 11 spots resulted in 24 protein identifications from the collated databases of *N. perurans* (Table 1), *Amoebozoa* and a discrete number of gram-negative bacteria chosen from the microbiome sequencing (Supplementary Table S2). The newly acquired culture showed the highest expression for the excised proteins, excluding spot 69 which was more upregulated in the attenuated culture. This protein may be considered a biomarker for reduced virulence in *N. perurans* as its expression shows that is markedly reduced in the virulent and newly acquired cultures.

Three proteins, glyceraldehyde-3 phosphate dehydrogenase (spot 314), chaperone protein (spot 283), putative TonB dependent receptor (spot 69) were associated only with *V. rumoiensis*, *Vibrio sp. C7* and *P. haloplanktis* respectively. The identity of the protein from spot 69 is aconitate hydratase AcnA, an iron sulphur regulatory protein, an essential enzyme in both the tricarboxylic acid cycle (TCA) and glyoxylate cycles and its presence is widely regarded as a biomarker for iron and redox stress^42^. Aconitate hydratase was identified from both *N. perurans* and the largely abundant *T. xiamenensis* (primarily of the attenuated microbiome) database therefore indicating this protein may be a bacterial protein derivative. The anaerobic bacterium, *Escherichia coli* maintains the use of isozyme AcnB in the TCA cycle, however upon growth in iron deficient and reactive oxygen species (ROS) stress, AcnB is deactivated and AcnA is expressed to relieve the metabolic block in the TCA cycle^43^. Aconitase AcnA elevated expression in the attenuated culture suggests long term cultured *N. perurans* withstands oxidative and/or iron deficiency stressors when isolated from the host. This is presumably from microbial community changes over time and plausibly as a result of non-optimal nutritional media. A putative TonB-dependent receptor from *P. haloplanktis* was also identified in this spot with a plausible role in signalling and siderophore activity. TonB – dependent receptors have been implicated in carbohydrate scavenging^44^, further suggesting *N. perurans* may face nutritional deficiencies during prolonged culture.

The proteins upregulated in the newly acquired culture can be attributed to cytoskeletal, oxidative and immunomodulatory roles. Numerous cytoskeleton associated proteins; actin cytoplasmic A3a isoform (spot 599), actin depolymerization factor-like-domain-containing protein (spot 487), tubulin beta chain (spot 314) and fragmin A (spot 283) were elevated in the newly acquired culture. Actin is a major cytoskeleton protein involved in motility, phagocytosis and tissue invasion in protozoan parasites^45–47^. Profilin (spot 582) and ADF-like-domain, an actin binding protein (ABP), were both found to be upregulated in the newly acquired culture. ABP’s are recruited for the regulation of dynamic actin remodelling during phagocytosis in *Entamoeba histolytica*^48^, a key process relating to *E. histolytica’s* pathogenesis. An allergic response to *N. perurans* has been postulated previously^49^ and profilin is commonly regarded as an allergen protein in many pollen and foods capable of inducing the production of IgE and IgM^50^. It is therefore plausible to think that this protein may also induce a similar response in the gills of Atlantic salmon.

Song et al.^51^ inoculated recombinant *Acanthamoeba* profilin intranasally in mice to determine the proteins potential for triggering allergic airway inflammation. Mice affected with the recombinant protein displayed classical signs of an allergic response; increased mucin production and hyperplasia of epithelial cells, accompanied by an elevated Th2 cytokine response. Apicomplexans require profilin for actin polymerisation and furthermore, profilin has been shown to be crucial in host tissue invasion, egress from the cell and stimulates host toll like receptors of the innate system^52^. Profilins role as an immunomodulator in AGD warrants extensive investigation based on its previous role as an immunogen in these protozoan and apicomplexan studies.

Elongation factor 1 alpha (EF1α) (spot 283) is vital for cytoskeleton integrity^53^ however it can serve numerous canonical functions in the cellular milieu hence being coined a moonlighting protein^54^. Elongation factors have been considered a virulence factor in both parasitic kinetoplastids and pathogenic bacteria as immunodominant antigens^55^. Macrophage deactivation occurs when *Leishmania donovani* EF1α binds to the hosts Src homology 2 domain containing protein tyrosine phosphatase-1 (SHP-1) and additionally inhibits inducible nitric oxide expression^56^. The protein’s potential as a potent vaccine antigen in *L*. *donovani* has been evaluated by Sabur et al.^55^ who generated liposomal forms of leishmanial elongation factor 1 vaccine and both the recombinant and truncated vaccine provided long term immunity in BALB/c mice against visceral leishmaniasis. Elongation factor thermo unstable (EF-Tu) (spot 283) was also upregulated in the newly acquired culture, and its identity was found in both *N. perurans* and *Pseudoaltermonas spp* databases. Ribosomal subunits as well as EF-Tu typically have roles in translation and this is conceivably the case here for *N. perurans*, however EF-Tu has also been reported as a cell surface protein that facilitates host cell attachment by binding of fibronectin^57^ and plasminogen^58^. Bacteria only express EF-Tu therefore, it appears *N. perurans* has either acquired this genetic material through lateral gene transfer from its commensal microbiome , a described occurrence associated with amoebae and bacteria^59^, or this protein is of bacterial origin entirely; a very plausible consideration due to the homologous EF-Tu in *Pseudoaltermonas spp.*

Proteins that support a function in oxidative stress were found to be upregulated in the newly acquired culture, specifically superoxide dismutase (SOD) (spot 487). A correlation between oxidative stress in hyperplastic lesions and late stage AGD has been shown for naïve smolts^60^. It has been previously reported that *E. histolytica* can directly interfere with host derived neutrophil ROS production and can also rely on a secondary ROS scavenging mechanism using Fe-superoxide dismutase’s^61^. The significantly reduced catalase enzyme in smolts displaying gill score of 2 from Marcos-Lopez et al.^60^.*,* study highlights the localised oxidative stress in the gill and suggests *N. perurans* is capable of tolerating the hostile environment by maintaining the expression of its own SOD.

Further protection from the host immune response is provided by heat shock proteins (HSP) that facilitate protein synthesis and simultaneously offer protective measures during episodes of oxidative, nutritional and heat stress in parasites^62^. The presence of HSP8 (spot 326) from *N. perurans,* HSP85 (spot 326) from *Perkinsela spp.* and HSP70 (spot 334) from *P. pallidum* suggests the role of oxidative protection is crucial in the newly acquired culture before adapatation slows its production in the virulent and attenuated cultures. A chaperone protein (spot 283) from *Vibrio sp. C7* was also found to be upregulated in the newly acquired isolate, potenially playing a similar role of oxidative protection. Malate dehydrogenase (spot 348) of *N. perurans* and glyceraldehyde-3 phosphate (spot 314), exclusively identified as *Vibrio rumoiensis*, are key enzymes involved in the TCA cycle that were upregulated in the newly acquired culture, highlighting the increased contribution of metabolism for *N. perurans* and its microbiome in the maintenance of virulence mechanisms.

Other metabolic proteins found to be upregulated in the newly acquired culture included citrate synthase (spot 283), ATP synthase (spot 511), ADP-ribosylation factor 4 and 1 (spot 511), and lipoxygenase (lox) homology domain-containing protein 1-like isoform X2 (spot 487). Citrate synthase is the first enzyme in the TCA cycle and ATP synthase is an essential nanomotor in the electron transport chain of the TCA cycle and thus, is responsible for the production of cellular ATP. Increased ATP production may be a virulence requisite for *N. perurans* as evidenced by the upregulation of these enzymes in the newly acquired culture with increased energy expenditure in the parasite due to replication and locomotion. Exploitation of ATP synthase is being considered as a drug target due to the novel subunits governing this protein in *T. gondii*^63^. This finding may offer a therapeutic avenue if *N. perurans* nanomotor shares homology with this ATP synthase structure. ADP-ribosylation factor 4 and 1 displayed a higher expression in the newly acquired culture with these GTP-binding proteins playing a role in protein tracking and cellular signalling^64^. Lipoxygenase constitutes a family of iron containing enzymes that serve as dioxygenases catalysts. In the nosocomial pathogen, *Pseudomonas aeruginosa*, lipoxygenase has been linked to increasing the bacterium’s persistence in the host, destroying the lipid membrane of epithelial cells^65^ and inhibiting the expression of chemokines ^66^.

The ATP binding cassette (ABC) membrane transporter proteins (spot 198) in protozoan parasites were best described as exporters of parasite waste, until *T. gondii* was shown to import host derived cholesterol^67^, which therefore suggests that transport may be bidirectional. They also represent an aspect of multi drug resistance in parasites^68^, therefore their characterisation is imperative in how future therapeutics may be effectively designed.

A characteristic of protozoan parasitic pathogenesis is the variation in antigenic representation to fulfil their parasitic lifecycle and gene management strategies remain at the forefront of this process. Chromatin is central to this role and histones are the building blocks of the nucleosomes that create chromatin structure. The elevation of histone H2B (spot 511) seen here in the newly acquired culture may highlight the underestimated role histones undertake in regulating transcription dependent on the parasites environment^69^. The most peculiar protein upregulated in the newly acquired culture was peptidase C53 family protein (spot 198), a protease homologue from pestiviruses, that would be typically located in the extracellular matrix as an excreted protease. The presence of a protease family in *N. perurans* however is not surprising as proteases assist in promoting pathogenesis through tissue destruction, invasion and facilitate immune evasion^70^. The identification of this protein in the cytoplasmic fraction suggests this protein remained in its pro-peptide form when it was isolated, a form seen when the protein is translocating towards the plasma membrane for extracellular secretion and release.

In conclusion, these findings suggest the attenuated *N. perurans* culture has a markedly reduced ability to establish disease in the host model as revealed by qPCR, histopathology and gill score. Microbial characterised by 16S rRNA gene sequencing successfully identified differences in diversity and relative abundances between the attenuated and virulent isolate. 2DE and LC–MS/MS successfully revealed the identity of 11 spots, of which 24 proteins were characterised and discussed here. Proteins in ten of the 11 spots were found to be significantly upregulated in the newly acquired culture and proteins in one spot, spot 69 containing bacterial protein derivatives; aconitate hydratase and TonB-dependent receptor proteins, was found to be significantly upregulated in the attenuated cohort. Many of the differential proteins followed a linear trend of expression, starting with the highest expression in the newly acquired, followed by intermediate expression in the virulent culture and the lowest expression in the attenuated culture. The current study focused on the soluble, cytoplasmic proteins of the *N. perurans* parasite and their association with pathogen virulence. This is supported by the upregulated proteins identified from the newly acquired culture which include the biological processes of cell cytoskeletal re-organisation, protein synthesis, oxidative stress and immunomodulation, all functions which ultimately suggest their involvement in acute AGD virulence. To further the identification of all proteins associated with AGD pathogenesis, future studies will investigate the proteomic profile of membrane-bound and extracellular proteins from the *N. perurans* cultures presented in this report.

## Materials and methods

All methods described were carried out in accordance with ARRIVE guidelines^71^ and relevant regulations. All experimental protocols were approved by the Health Products Regulatory Authority (HPRA) in Ireland under project authorisation number AE 19137/P002 following the Animals Scientific Procedures Act 1986 (Directive 2010/63/EU transposed into Irish law by S.I. No 543 of 2012).

### *Neoparamoeba perurans* isolation and culture

*N. perurans* trophozoites were isolated from AGD infected Atlantic salmon located on a commercial farm in the west of Ireland. Trophozoites were collected by swabbing AGD infected gills and placing swabs in 0.2 µm filtered sterile seawater for 4 h. This combined mixture was subsequently plated and maintained xenically at 16 °C on marine yeast agar plates (MYA; 0.01% malt, 0.01% yeast, 2% Bacto Agar, sterile sea water at 30 ppt salinity) overlaid with 7 mL of 0.2 µm filtered sea water^72^.

Inoculated plates were washed weekly with 7 mL of sterile seawater to control bacterial growth. The amoebae were sub-cultured weekly by transferring free-floating cells to fresh MYA plates. Confirmation of *N. perurans* identity was performed using real time PCR as previously described by Downes et al*.*^32^. Parasites from cultures established at 3-years, 1-year and 70 days are referred to as attenuated, virulent and newly acquired, respectively. The attenuated culture of amoeba was established in October 2015, while the virulent culture was propagated for 1 year prior to inoculation of naïve smolts. These two cultures were used in the in vivo challenge trial. The newly acquired culture was propagated for 70 days prior to harvesting for proteomic analysis.

### In vivo challenge trial

Naïve Atlantic salmon smolts (n = 120) weighing approximately 120 g were transported from a commercial hatchery to the animal housing unit in Galway-Mayo Institute of Technology. Fish were divided into three cohorts, namely the control cohort (n = 40), the attenuated cohort (n = 40) and virulent cohort (n = 40). These cohorts were distributed into six circular 1000 L (n = 20) circular tanks and maintained for a 2-week acclimation period prior to inoculation. Tanks were monitored for dissolved oxygen, salinity and water quality. Tanks were maintained at 16 °C and salinity of 30 ppt. All fish used in this study were fed daily to satiation using a commercial food pellet.

Fish were transferred to a 300 L saltwater bath at 30 ppt and temperature maintained at 16 °C for inoculation with the parasite for 4 h. The control group were placed in a 300 L saltwater bath, minus the addition of the parasite, serving as the negative control. The attenuated cohort was challenged with 2000 cells/L, achieved by cell count using a Sedgewick rafter counting chamber (API Supplies), of a 3-year old *N*. *perurans* culture that was maintained since October 2015. The virulent cohort was challenged with 2000 cells/L of a 1-year old *N perurans* culture that was maintained since October 2019. After the bathing period, fish were placed back into their respective 1000 L tanks for the duration of the trial.

### Validation of virulence

Six fish from each cohort (n = 18) were removed at 0, 7, 14 and 21-days post infection (dpi) and euthanized in an overdose of anaesthetic containing 400 mg/L of tricaine methane sulfonate (MS-222) for lethal sample collection. Gross macroscopic gill scores were assessed according to Taylor et al.^73^. Gill samples from the first gill arch were excised for histology and quantitative polymerase chain reaction (qPCR) detection of *N. perurans.* Samples for histological examination were placed into 10% neutral buffered formalin (Sigma-Aldrich) and were processed by the Agri-Food & Biosciences institute (AFBI Stormont). Image analysis of histology samples was undertaken using Olympus cellSens Standard ver 1.12 (http://www.olympus-lifescience.com/en/software/cellsens/) and Image J (National Institutes of Health, https://imagej.nih.gov/ij/download.html) software. Samples for qPCR detection of *N. perurans* were excised, flash frozen and analysed as described by Downes et al.^32^.

### RNA sequencing of *N. perurans* microbiome

#### DNA Extraction from *N. perurans* cultures

DNA was extracted using the AllPrep DNA/RNA Mini Kit (QIAGEN, Hilden, Germany) from the attenuated and virulent *N. perurans* cultures in triplicate. Three plates of the attenuated and virulent cultures were individually prepped for DNA extraction by mechanically scraping cells from MYA plates into a 50 mL falcon tube, yielding a suspension of adherant and floating cells in seawater. The suspensions were briefly vortexed to homogenise. One mL of each *N. perurans* cell suspension was added to 600 μL of RLT lysis buffer followed by vortexing for 10 min. Fresh lysozyme dilution (20 μL of 10 mg/mL) was added to each tube, prior to 30 min incubation at 37 °C on a shaker platform. Subsequently, 10 μL of Proteinase K (> 600 mAU/ml; QIAGEN, Hilden, Germany) was added into each tube followed by 30 min incubation at room temperature. The rest of the DNA extraction was performed following manufacturer’s instructions (AllPrep DNA/RNA Mini Kit [QIAGEN, Hilden, Germany]). All DNA concentrations after the extraction processing were measured using Qubit dsDNA BR Assay Kit (ThermoFisher Scientific, Paisley, United Kingdom), following its instructions.

#### 16S rRNA gene sequencing in Illumina

The sequencing targeted the V4 and ITS1-spanning regions from 16S rRNA genes in the DNA samples using the primers 515F (5′-GTGCCAGCMGCCGCGGTAA-3′) and 806R (5′-GGACTACHVGGGTWTCTAAT-3′)^74^. All sequencing was carried out in FISABIO’s facilities (FISABIO / Avda. de Catalunya, 21/46020 Valencia, Spain). PCR reaction tubes contained (25 µL final volume): 12.5 ng (genomic DNA), 0.2 µM (final concentration) 515F primer, 0.2 µM 806R, and 12.5 µL 2 × KAPA HiFi Hot Start Ready Mix (0.5 U per 25 µL reaction; Roche). All PCR reactions followed the same program: 1) 95 °C for 3 min, 2) 25 cycles containing: 95 °C for 30 s 55 °C for 30 s and 72 °C for 30 s; 3) 72 °C for 30 s. After a PCR clean-up on each sample, Illumina sequencing adapters were added using Nextera XT Index Kit (Cambridge, United Kingdom) following instructions from the company. Prior to DNA quantification (Agilent Technologies 2100 Bioanalyzer), a second PCR cleaning-up was done. A DNA normalization (to 4 nM) protocol was done, followed by libraries pooling with unique indices. Pools were denatured (using 0.2 N of NaOH), diluted and heat denatured before sequencing. Resulting amplicons were sequenced (Illumina MiSeq platform^75^ using 5% PhiX as an internal control.

#### Sequencing analyses

All sequenced demultiplexed FASTQ data from samples were analysed using QIIME 2.2019.10 (Quantitative Insight Into Microbial Ecology) following the “Moving Pictures” tutorial workflow from the official QIIME 2 website (https://docs.qiime2.org/2019.10/tutorials/moving-pictures/)^76^. Raw sequences were imported using the “Casava 1.8 paired-end demultiplexed fastq” procedure. They were assembled and quality filtered at a phred score limit of Q25. Resulting files were denoised using the Dada2 command. Sequences were aligned and clustered based on a 97% identity, later each cluster was taxonomically classified using the Greengenes database 13–8^77^. Taxonomic bar plots were visualised and saved from the QIIME 2 View webpage (https:// view.qiime2.org/).

### Protein extraction of *N. perurans* cultures

The newly acquired, virulent, and attenuated cultures of *N. perurans* were harvested in isolation of each other to avoid cross contamination. Confluent cultures of *N. perurans* were harvested by mechanical scraping followed by centrifugation at 1000 × g for 10 min at 4 °C. Cell pellets were resuspended in lysis buffer (7 M urea, 2% w/v CHAPS and 50 mM DTT) with HALT protease inhibitor cocktail (Thermo Fisher). Samples were incubated on ice for 30 min and sonicated at 35 Hz on ice for 3 cycles of 30 s “pulse on” and 15 s “pulse off”. Samples were centrifuged to pellet the cell debris at 12, 000 × g at 4 °C for 10 min and the supernatant was transferred to a fresh 1.5 mL microcentrifuge tube for further removal of residual seawater. Each supernatant received 20% trichloroacetic acid TCA in acetone and was centrifuged for 30 min at 4 °C at 12,000 × *g*. The pellet, containing precipitated proteins, was washed twice in 100% acetone and left to air dry before solubilisation in rehydration buffer 1 (7 M urea, 2 M thiourea, 1% ASB-14, 40 Mm Tris, 0.001% Bromophenol Blue, 2% Bio-Lyte [Bio-Rad, Hercules, CA]). Samples were incubated for 60 min at room temperature before determining protein concentration using the RC/DC kit (Bio-Rad).

### Two-dimensional electrophoresis

The soluble protein fraction from the newly acquired, virulent and attenuated *N. perurans* cultures were analysed in triplicate using 2D electrophoresis. Triplicate immobilised pH gradient strips (IPG, pH 4–7, 11 cm) were passively rehydrated overnight with 120 µg of protein in a final volume of 200 µL of re-hydration buffer 1. The strips were focused in a PROTEAN i12 IEF system (Bio-Rad) at a current limit of 50 Amp/IPG strip using a step voltage gradient (250 V for 20 min, stepped up to 8000 V maximum for 1 h; 26,000 V-h total) at 20 °C. Prior to the second dimension IPG strips were equilibrated in a reducing buffer (6 M urea, 0.375 M Tris–HCl pH 8.8, 2% (w/v) SDS, 20% (v/v) glycerol, 2% (w/v) dithiothreitol ) for 20 min and subsequently placed in an alkylating buffer (6 M urea, 0.375 M Tris–HCL pH 8.8, 2% (w/v) SDS, 20% (v/v) glycerol, 2.5% iodoacetamide) for 20 min. The second dimension was performed using AnykD Criterion TGX Precast gel in the Criterion Dodeca cell (Bio-Rad, CA) where IPG strips were electrophoresed for 40 min at 200 V. Gels were stained using QC Colloidal Coomassie (Bio-Rad) overnight and destained by washing in deionized water in triplicate, followed by incubation in wash buffer (10% ethanol [v/v], 7.5% acetic acid [v/v]).

### Gel image analysis

Gels images were acquired using the Gel Doc EZ Gel Documentation System (Bio-Rad, CA) and images analysed using the SameSpots 5.1.0.0 software (TotalLab, Non-linear Dynamics, UK). Triplicate gels used to analyse the proteins extracted from the newly acquired, virulent and attenuated cultures were automatically aligned, and normalization of spots was performed to detect differentially expressed proteins. The software normalises volumes of each spot and detects differential proteins based on a difference in spot intensity using the ‘between subject’ experimental design, which performs an ANOVA calculation. Differential spots were chosen for further analysis based on maximum fold change of ≥ 2 and ANOVA statistical significance (*p* < 0.05) using the software’s statistical analysis function.

### Spot preparation and mass spectrometry

Enzymatic in-gel digestion was performed as described by Shevchenko et al.^78^. Gels were rinsed with deionised water and spots of interest were excised and cut into pieces using a sterile scalpel. Gel pieces were microcentrifuged to collect excess water, destained with 100 µL of 100 mM ammonium bicarbonate/acetonitrile (1:1, v/v) and incubated for 30 min with vortexing every 10 min. Following the addition of ammonium bicarbonate, 500 µL of neat acetonitrile (ACN) was added and incubated at room temperature until gel pieces were destained and decreased in size. The gel pieces were dried and rehydrated with trypsin buffer overnight at 37 °C (13 ng/µl trypsin in 10 mM ammonium bicarbonate containing 10% [v/v] ACN on a thermomixer at 350 rpm.

To terminate enzymatic digestion, 1% acetic acid (AA) was added to the samples. Peptides were desalted using C18 stage tips with Equilibration Buffer (0.1% trifluoroacetic acid [TFA] in MS grade water) and Elution Buffer (50% ACN 0.1% TFA in MS grade water). After drying by vacuum centrifugation, peptides were acidified by AA, desalted with C18 STAGE tips^79^, and resuspended in 2.5% ACN, 0.5% AA. Peptide fractions were analysed on a quadrupole Orbitrap (Thermo Scientific Q Exactive Hybrid Quadrupole-Orbitrap Mass Spectrometer) mass spectrometer equipped with a reversed-phase NanoLC UltiMate 3000 HPLC system (Dionex LC Packings, now Thermo Scientific). Peptide samples were loaded onto C18 reverse phase columns (10 cm length, 75 µm inner diameter) and eluted with a linear gradient from 1 to 27% buffer B containing 0.5% AA 97.5% ACN in 58 min at a flow rate of 250 nL/min. The injection volume was 5 μL.

Raw data from the Orbitrap Q-Exactive (Thermo Scientific Q Exactive Hybrid Quadrupole-Orbitrap Mass Spectrometer) was processed using MaxQuant version 1.6.6.0 for identification of proteins^80^, incorporating the Andromeda search engine and MaxQuants contaminants fasta file^31^. To identify peptides and proteins, MS/MS spectra were matched to a combined custom database comprised of *N. perurans* (20,887 proteins [v2, 07/08/2019, CSIRO]); UniProt reference proteome databases of *Amoebozoa* (109,415 proteins) and bacteria selected from the microbiome analysis (148,582 proteins) as well as a database of *Paramoeba* proteins from a taxonomy search on UniProtKB (5001 proteins). All databases except for *N. perurans* were downloaded on May 8th 2020 from UniProt^81^ and further information on individual species protein counts are in Supplementary Tables S3, S4 and S5.

All searches were performed with tryptic specificity allowing two missed cleavages. The database searches were performed with carbamidomethyl (C) as fixed modification and acetylation (protein N terminus) and oxidation (M) as variable modifications. Mass spectra were searched using the default setting of MaxQuant with a false discovery rate of 1% at the peptide and protein level.

## Supplementary information

Supplementary Information.

## Data Availability

All data supporting this study is included in the results section of the manuscript, the supplementary material or openly available in public databases. The mass spectrometry proteomics data have been deposited to the ProteomeXchange Consortium via the PRIDE partner^82^ repository with the dataset identifier PXD022328.
