# Author Correction: Comparative proteomic profiling of newly acquired, virulent and attenuated *Neoparamoeba perurans* proteins associated with amoebic gill disease

**DOI:** 10.1038/s41598-021-01760-y

**Published:** 2021-11-09

**Authors:** Kerrie Ní Dhufaigh, Eugene Dillon, Natasha Botwright, Victor Birlanga, Anita Talbot, Ian O’Connor, Eugene MacCarthy, Orla Slattery

**Affiliations:** 1 Marine and Freshwater Research Centre, Galway-Mayo Institute of Technology, Co. Galway, Ireland; 2 Conway Institute, University College Dublin, Co. Dublin, Ireland; 3 CSIRO Agriculture and Food, Livestock & Aquaculture, Queensland Biosciences Precinct, 306 Carmody Road, Brisbane, QLD 4067 Australia; 4 Microbiology Department, School of Natural Sciences, National University of Ireland Galway, University Road, Co. Galway, Ireland; 5 Department of Biopharmaceutical and Medical Science, Galway-Mayo Institute of Technology, Co. Galway, Ireland

Correction to: *Scientific Reports* 10.1038/s41598-021-85988-8, published online 25 March 2021

Victor Birlanga was omitted from the author list in the original version of this Article.

The Author Contributions section now reads:

“A.T, I.O.C, E.MC, O.S designed the experiments. K.N.D collected and maintained amoeba isolates and performed the protein extractions. K.N.D and O.S analysed and processed the raw mass spectrometry data. E.D provided statistical input on the mass spectrometry analysis. N.B provided the *N. perurans* protein database. V.B extracted *N. perurans* DNA, developed the OTU graph and aided in the results interpretation. K.N.D and O.S wrote the manuscript and critical revision was provided by A.T, I.O.C, E.MC, N.B and E.D.”

The original Article and the Supplementary Information file that accompanies the article have been corrected.

