# Supplementary material for: Comparative proteomic profiling of newly acquired, virulent and attenuated Neoparamoeba perurans proteins associated with amoebic gill disease
Source: Sci Rep. 2021 Mar 25;11:6830. doi: 10.1038/s41598-021-85988-8 (PMC7994405; doi:10.1038/s41598-021-85988-8)
Supplement: Supplementary file 1 — Supplementary Information. [file 41598_2021_85988_MOESM1_ESM.docx]

**Comparative proteomic profiling of newly acquired, virulent and attenuated *Neoparamoeba perurans* proteins associated with amoebic gill disease**

Kerrie Ní Dhufaigh^a*^, Eugene Dillon^b^, Natasha Botwright^c^, Victor Birlanga^d^, Anita Talbot^a^, Ian O’Connor^a^, Eugene MacCarthy^a^, Orla Slattery^e^

^a^Marine and Freshwater Research Centre, Galway-Mayo Institute of Technology, Co. Galway, Ireland

^b^Conway Institute, University College Dublin, Co. Dublin, Ireland

^c^CSIRO Agriculture and Food, Livestock & Aquaculture, Queensland Biosciences Precinct, 306 Carmody Road, Brisbane, Queensland 4067, Australia

^d^Microbiology Department, School of Natural Sciences, National University of Ireland Galway, University Road, Co. Galway, Ireland

^e^Department of Biopharmaceutical and Medical Science, Galway-Mayo Institute of Technology, Co. Galway, Ireland

*Corresponding author. Marine and Freshwater Research Centre, Galway-Mayo Institute of Technology, Co. Galway, Ireland

Email: [kerrie.nidhufaigh@research.gmit.ie](mailto:kerrie.nidhufaigh@research.gmit.ie)

**Supplementary Table S1:** *N. perurans* gene bank identifications and associated spot number

| GenBank  IDs | Spot  number |
| --- | --- |
|  |  |
| **MT419751** | 511 |
| **MT419752** | 511 |
| **MT419753** | 511 |
| **MT419755** | 326 |
| **MT419754** | 198 |
| **MT419757** | 198 |
| **MT419756** | 599 |
| **MT419758** | 487 |
| **MT419759** | 487 |
| **MT419760** | 487 |
| **MT419762** | 582 |
| **MT419763** | 582 |
| **MT419761** | 348 |
| **MT419767** | 283 |
| **MT419765** | 283 |
| **MT683515** | 283 |
| **MT683516** | 283 |
| **MT419769** | 69 |

**Supplementary Table S2:** 2D spots with significant (p ≤0.05) fold changes from the newly acquired *N. perurans* culture identified by LC-MS/MS. A large collated database containing *N. perurans* microbiome, Amoebozoa and *N. perurans* proteins was used in the database search of MaxQuant. Several proteins that are shared between *N. perurans* and species found in the bacteria and Amoebozoa database are distinguished in the organism header. Genes are related to non-*N. perurans* identifications

| **Genes** | **Spot number** | **Estimated MW kDa** | **Estimated pI** | **Fold change** | ***P*- value** | **Fasta header** | **Protein identification** | **Organism** | **No Peptides matched** | **Main Biological function** |
| --- | --- | --- | --- | --- | --- | --- | --- | --- | --- | --- |
|  |  |  |  |  | ≤0.05 |  |  |  |  |  |
| - | 511 | 20 | 5.61 | 4.6 | 0.005 | PPER_00018276-RA | ATP synthase subunit mitochondrial-like | *N. perurans* | 2 | Metabolism |
| ACA1_265860 | 511 | 20 | 5.61 | 4.6 | 0.005 | PPER_00019391-RA;  L8H4F8 | Histone H2B | *N. perurans; Acanthamoeba castellanii str. Neff* | 2 | Protein synthesis |
| EHI_073470 | 511 | 20 | 5.61 | 4.6 | 0.005 | PPER_00018720-RA; C4LXL1 | ADP-ribosylation factor 4 and 1; ADP-ribosylation factor ,putative | *N. perurans; Entamoeba histolytica* | 2 | Cellular signalling |
| XU18_0391 | 326 | 40 | 5.55 | 3 | 0.042 | PPER_00010306-RA; A0A0L1L0Q5 | Heat shock protein 8; Heat shock protein 85 | *N. perurans; Perkinsela sp. CCAP 1560/4* | 3 | Stress response |
| - | 198 | 70 | 4.45 | 2.5 | 9.55E-05 | PPER_00013769-RA | Peptidase C53 family protein | *N. perurans* | 4 | Immune evasion |
| - | 198 | 70 | 4.45 | 2.5 | 9.55E-05 | PPER_00000416-RA | Carbohydrate ABC transporter substrate-binding protein | *N. perurans* | 3 | Metabolism |
| ACA1_219820 | 599 | 16 | 6.12 | 4.4 | 1.10E-04 | PPER_00017761-RA; L8GT00 | Actin, cytoplasmic A3a isoform;  Actin-1, putative | *N. perurans; Acanthamoeba castellanii str. Neff* | 8 | Cytoskeleton |
| - | 487 | 21 | 5.95 | 2.1 | 0.003 | PPER_00012939-RA | Cu-Zn Superoxide dismutase | *N. perurans* | 2 | Oxidative response |
| - | 487 | 21 | 5.95 | 2.1 | 0.003 | PPER_00019832-RA | ADF-like domain-containing protein | *N. perurans* | 2 | Cytoskeleton |
| - | 487 | 21 | 5.95 | 2.1 | 0.003 | PPER_00020879-RA | Lipoxygenase (lox) homology domain-containing protein 1 | *N. perurans* | 2 | Cellular signalling |
| - | 582 | 17 | 6.08 | 7.2 | 6.25E-04 | PPER_00013951-RA | Profilin conserved site domain-containing protein | *N. perurans* | 6 | Cytoskeleton, immunomodulation |
| - | 582 | 17 | 6.08 | 7.2 | 6.25E-04 | PPER_00018653-RA | Profilin allergen | *N. perurans* | 6 | Cytoskeleton, immunomodulation |
| - | 348 | 36 | 5.61 | 5.2 | 0.007 | PPER_00013918-RA | Malate dehydrogenase | *N. perurans* | 4 | Metabolism |
| PPL_03556 | 334 | 39 | 6.14 | 6.2 | 0.007 | D3B545 | Hsc70 protein | *Polysphondylium pallidum* (strain ATCC 26659 / Pp 5 / PN500) | 4 | Stress response |
| PROFUN_09625 | 314 | 41 | 5.48 | 3.5 | 0.041 | A0A2P6MNZ1 | Tubulin beta chain | *Planoprotostelium fungivorum* | 3 | Cytoskeleton |
| A1QC_14775 | 314 | 41 | 5.48 | 3.5 | 0.041 | A0A1E5E4F2 | Glyceraldehyde-3-phosphate dehydrogenase | *Vibrio rumoiensis 1S-45* | 2 | Metabolism |
| tuf | 283 | 45 | 5.36 | 1.9 | 0.033 | A0A0A7ECJ2 | Elongation factor Tu | *Pseudoalteromonas piratica* | 6 | Cytoskeleton, immunomodulation |
| - | 283 | 45 | 5.36 | 1.9 | 0.033 | PPER_00019602-RA | Fragmin A | *N. perurans* | 3 | Cytoskeleton |
| JCM19233_3548 | 283 | 45 | 5.36 | 1.9 | 0.033 | A0A090RBI3 | Chaperone protein DnaK | *Vibrio sp. C7* | 2 | Multifunctional, stress response |
| - | 283 | 45 | 5.36 | 1.9 | 0.033 | PPER_00020852-RA; W5RWF5 | Elongation factor 1; Elongation factor 1-alpha (Fragment) | *N. perurans; Paramoeba pemaquidensis* | 2 | Cytoskeleton |
| - | 283 | 45 | 5.36 | 1.9 | 0.033 | PPER_00013476-RA | Component of cytosolic 80S ribosome and 60S large subunit | *N. perurans* | 2 | Translation |
| - | 283 | 45 | 5.36 | 1.9 | 0.033 | PPER_00013717-RA | Citrate synthase active | *N. perurans* | 2 | Metabolism |
| TH44_03405 | 69 | 142 | 4.5 | 3.3 | 0.018 | PPER_00005663-RA; A0A367XHX2 | Aconitate hydratase | *N. perurans; Thalassospira xiamenensis* | 2 | Oxidative stress |
| PSHAa1352 | 69 | 142 | 4.5 | 3.3 | 0.018 | Q3IL25 | Putative TonB-dependent receptor | *Pseudoalteromonas haloplanktis* (strain TAC 125) | 3 | Receptor |

**Supplementary Table S3:** Bacterial reference proteome downloaded on the 8^th^ of May 2020 from UniProt .

| **Proteome ID** | **Organism** | **Organism ID** | **Protein count** | **BUSCO** | **CPD** | **Genome representation (RefSeq)** |
| --- | --- | --- | --- | --- | --- | --- |
| UP000198862 | Pseudoalteromonas denitrificans DSM 6059 (Strain: DSM 6059) | 1123010 | 5332 | C:98.7%[S:98.2%,D:0.4%],F:0.4%,M:0.9%,n:452 | Close to Standard | full |
| UP000033452 | Pseudoalteromonas rubra (Strain: S2471) | 43658 | 4390 | C:98.7%[S:98.5%,D:0.2%],F:0%,M:1.3%,n:452 | Standard | full |
| UP000033511 | Pseudoalteromonas piscicida (Strain: S2040) | 43662 | 4134 | C:97.1%[S:96.7%,D:0.4%],F:0.2%,M:2.7%,n:452 | Close to Standard | full |
| UP000179786 | Pseudoalteromonas amylolytica (Strain: JW1) | 1859457 | 4015 | C:99.3%[S:98.9%,D:0.4%],F:0.4%,M:0.2%,n:452 | Standard | full |
| UP000030341 | Pseudoalteromonas piratica (Strain: OCN003) | 1348114 | 3997 | C:99.8%[S:99.3%,D:0.4%],F:0%,M:0.2%,n:452 | Standard | full |
| UP000033664 | Pseudoalteromonas ruthenica (Strain: S3137) | 151081 | 3470 | C:98.5%[S:98%,D:0.4%],F:0.2%,M:1.3%,n:452 | Standard | full |
| UP000061457 | Pseudoalteromonas phenolica (Strain: KCTC 12086) | 161398 | 4164 | C:98.9%[S:98.2%,D:0.7%],F:0.4%,M:0.7%,n:452 | Standard | full |
| UP000006843 | Pseudoalteromonas haloplanktis (strain TAC 125) (Strain: TAC 125) | 326442 | 3484 | C:99.3%[S:98.9%,D:0.4%],F:0.2%,M:0.4%,n:452 | Standard | full |
| UP000016487 | Pseudoalteromonas citrea DSM 8771 (Strain: DSM 8771) | 1117314 | 4457 | C:99.6%[S:99.3%,D:0.2%],F:0.2%,M:0.2%,n:452 | Standard | full |
| UP000194841 | Pseudoalteromonas ulvae | 107327 | 3964 | C:99.3%[S:98.9%,D:0.4%],F:0%,M:0.7%,n:452 | Standard | full |
| UP000076643 | Pseudoalteromonas luteoviolacea DSM 6061 (Strain: DSM 6061) | 1365250 | 5008 | C:99.6%[S:98.9%,D:0.7%],F:0%,M:0.4%,n:452 | Standard | full |
| UP000007127 | Thalassospira xiamenensis M-5 = DSM 17429 (Strain: M-5) | 1123366 | 4340 | C:98.2%[S:98.2%,D:0%],F:0.5%,M:1.4%,n:221 | Standard | full |
| UP000252266 | Thalassospira xiamenensis (Strain: IB13) | 220697 | 4080 | C:96.4%[S:96.4%,D:0%],F:0.9%,M:2.7%,n:221 | Standard | full |
| UP000253064 | Thalassospira xiamenensis (Strain: S27-11) | 220697 | 4028 | C:97.3%[S:97.3%,D:0%],F:0.5%,M:2.3%,n:221 | Standard | full |
| UP000219068 | Thalassospira xiamenensis (Strain: USBA 78) | 220697 | 5070 | C:98.2%[S:95.9%,D:2.3%],F:0.5%,M:1.4%,n:221 | Outlier | full |
| UP000007463 | Fluviicola taffensis (strain DSM 16823 / NCIMB 13979 / RW262) (Strain: DSM 16823 / RW262 / RW262) | 755732 | 4016 | C:97.1%[S:96.6%,D:0.5%],F:1.6%,M:1.4%,n:443 | Close to Standard | full |
| UP000000584 | Vibrio cholerae serotype O1 (strain ATCC 39315 / El Tor Inaba N16961) (Strain: ATCC 39315 / El Tor Inaba N16961) | 243277 | 3782 | C:98.9%[S:98.5%,D:0.4%],F:0%,M:1.1%,n:452 | Standard | full |
| UP000000537 | Aliivibrio fischeri (strain ATCC 700601 / ES114) (Vibrio fischeri) (Strain: ATCC 700601 / ES114) | 312309 | 3813 | C:99.3%[S:99.1%,D:0.2%],F:0.7%,M:0%,n:452 | Standard | full |
| UP000232179 | Vibrio sp. HA2012 (Strain: HA2012) | 1971595 | 3382 | C:99.8%[S:99.6%,D:0.2%],F:0%,M:0.2%,n:452 | Standard | full |
| UP000235640 | Vibrio sp. 10N.286.49.C2 (Strain: 10N.286.49.C2) | 1880856 | 4338 | C:99.8%[S:98.7%,D:1.1%],F:0%,M:0.2%,n:452 | Standard | full |
| UP000009100 | Vibrio tasmaniensis (strain LGP32) (Vibrio splendidus (strain Mel32)) (Strain: LGP32) | 575788 | 4420 | C:99.1%[S:97.6%,D:1.5%],F:0.9%,M:0%,n:452 | Standard | full |
| UP000094165 | Vibrio genomosp. F6 str. FF-238 (Strain: FF-238) | 1191298 | 3650 | C:98.7%[S:98.5%,D:0.2%],F:0.4%,M:0.9%,n:452 | Standard | full |
| UP000094070 | Vibrio rumoiensis 1S-45 (Strain: 1S-45) | 1188252 | 2981 | C:99.1%[S:99.1%,D:0%],F:0%,M:0.9%,n:452 | Close to Standard | full |
| UP000002943 | Vibrio caribbeanicus ATCC BAA-2122 (Strain: ATCC BAA-2122) | 796620 | 4023 | C:99.3%[S:99.3%,D:0%],F:0.2%,M:0.4%,n:452 | Standard | full |
| UP000184608 | Vibrio aerogenes CECT 7868 (Strain: CECT 7868) | 1216006 | 4565 | C:99.3%[S:99.1%,D:0.2%],F:0.7%,M:0%,n:452 | Standard | full |
| UP000281112 | Vibrio sp. LJC006 (Strain: LJC006) | 2487322 | 4277 | C:100%[S:99.3%,D:0.7%],F:0%,M:0%,n:452 | Standard | full |
| UP000016567 | Vibrio azureus NBRC 104587 (Strain: NBRC 104587) | 1219077 | 4147 | C:99.6%[S:98.9%,D:0.7%],F:0.4%,M:0%,n:452 | Standard | full |
| UP000002493 | Vibrio parahaemolyticus serotype O3:K6 (strain RIMD 2210633) (Strain: RIMD 2210633) | 223926 | 4821 | C:99.6%[S:98.7%,D:0.9%],F:0.2%,M:0.2%,n:452 | Standard | full |
| UP000193432 | Vibrio sp. qd031 (Strain: qd031) | 1603038 | 3553 | C:98.5%[S:98%,D:0.4%],F:0%,M:1.5%,n:452 | Standard | full |
| UP000269041 | Vibrio pectenicida (Strain: CAIM 594) | 62763 | 3823 | C:99.1%[S:94.7%,D:4.4%],F:0%,M:0.9%,n:452 | Standard | full |
| UP000037515 | Vibrio nereis (Strain: DSM 19584) | 693 | 3660 | C:100%[S:99.6%,D:0.4%],F:0%,M:0%,n:452 | Standard | full |
| UP000189475 | Vibrio palustris (Strain: CECT 9027) | 1918946 | 3328 | C:99.6%[S:98.2%,D:1.3%],F:0.2%,M:0.2%,n:452 | Standard | full |
| UP000029994 | Vibrio navarrensis (Strain: ATCC 51183) | 29495 | 3643 | C:99.8%[S:99.1%,D:0.7%],F:0%,M:0.2%,n:452 | Standard | full |
| UP000016895 | Vibrio nigripulchritudo (Strain: SnF1) | 28173 | 5540 | C:100%[S:99.8%,D:0.2%],F:0%,M:0%,n:452 | Standard | full |
| UP000003627 | Vibrio sp. (strain N418) (Strain: N418) | 701176 | 4028 | C:99.6%[S:98.2%,D:1.3%],F:0.4%,M:0%,n:452 | Standard | full |
| UP000198854 | Vibrio xiamenensis (Strain: CGMCC 1.10228) | 861298 | 4859 | C:100%[S:98.9%,D:1.1%],F:0%,M:0%,n:452 | Standard | full |

**Supplementary Table S4:** Amoebozoa reference proteome downloaded on the 8^th^ of May 2020 from UniProt

| **Proteome ID** | **Organism** | **Organism ID** | **Protein count** | **BUSCO** | **CPD** | **Genome representation (RefSeq)** |
| --- | --- | --- | --- | --- | --- | --- |
| UP000001064 | Dictyostelium purpureum (Slime mold) (Strain: QSDP1) | 5786 | 12347 | C:92.1%[S:89.1%,D:3%],F:2%,M:5.9%,n:303 | Standard | full |
| UP000002195 | Dictyostelium discoideum (Slime mold) (Strain: AX4) | 44689 | 12746 | C:96%[S:92.4%,D:3.6%],F:0.7%,M:3.3%,n:303 | Standard | full |
| UP000001396 | Polysphondylium pallidum (strain ATCC 26659 / Pp 5 / PN500) (Heterostelium pallidum) (Strain: ATCC 26659 / Pp 5 / PN500) | 670386 | 12351 | C:92.1%[S:92.1%,D:0%],F:3.6%,M:4.3%,n:303 | Standard | full |
| UP000001926 | Entamoeba histolytica (Strain: ATCC 30459 / HM-1:IMSS) | 5759 | 7959 | C:65.7%[S:54.1%,D:11.6%],F:6.9%,M:27.4%,n:303 | Standard | full |
| UP000241769 | Planoprotostelium fungivorum (Strain: Jena) | 1890364 | 16856 | C:94.4%[S:87.5%,D:6.9%],F:2%,M:3.6%,n:303 | Outlier | full |
| UP000014680 | Entamoeba invadens IP1 (Strain: IP1) | 370355 | 9857 | C:53.8%[S:49.5%,D:4.3%],F:4.3%,M:41.9%,n:303 | Close to Standard | full |
| UP000076078 | Tieghemostelium lacteum (Strain: TK) | 361077 | 10208 | C:93.4%[S:92.4%,D:1%],F:2.3%,M:4.3%,n:303 | Outlier | full |
| UP000011083 | Acanthamoeba castellanii str. Neff (Strain: Neff) | 1257118 | 14939 | C:81.5%[S:77.6%,D:4%],F:5.9%,M:12.5%,n:303 | Close to Standard | full |
| UP000007797 | Cavenderia fasciculata (strain SH3) (Slime mold) (Dictyostelium fasciculatum) (Strain: SH3) | 1054147 | 12152 | C:92.1%[S:90.4%,D:1.7%],F:2.3%,M:5.6%,n:303 | Standard | full |

**Supplementary Table S5:** Paramoeba taxonomy search on UniProt KB downloaded on 8^th^ of May 2020.

| **Organism** | **Total protein Count** | **Taxon ID** |
| --- | --- | --- |
| Candidatus Syngnamydia salmonis | 9 | 504270 |
| Paramoeba aestuarina | 1 | 180227 |
| Paramoeba aparasomata | 39 | 2583407 |
| Paramoeba branchiphila | 1 | 308475 |
| Paramoeba eilhardi | 1 | 200891 |
| Paramoeba invadens | 1 | 1321612 |
| Paramoeba karteshi | 2 | 2583406 |
| Paramoeba pemaquidensis | 62 | 180228 |
| Paramoeba perurans | 2 | 437603 |
| Perkinsela sp. CCAP 1560/4 | 4883 | 1314962 |
